# The N-Formyl Peptide Receptor 2 (FPR2) Agonist MR-39 Improves Ex Vivo and In Vivo Amyloid Beta (1–42)-Induced Neuroinflammation in Mouse Models of Alzheimer’s Disease

**DOI:** 10.1007/s12035-021-02543-2

**Published:** 2021-09-01

**Authors:** Ewa Trojan, Kinga Tylek, Nicole Schröder, Iris Kahl, Lars-Ove Brandenburg, Margherita Mastromarino, Marcello Leopoldo, Agnieszka Basta-Kaim, Enza Lacivita

**Affiliations:** 1 Department of Experimental Neuroendocrinology, Immunoendocrinology Laboratory, Maj Institute of Pharmacology Polish Academy of Sciences, 12 Smętna Str, 31-343 Kraków, Poland; 2 Institute of Anatomy, Rostock University Medical Center, Rostock, Germany; 3 Department of Anatomy and Cell Biology, RWTH Aachen University, Aachen, Germany; 4 Department of Pharmacy–Drug Sciences, University of Bari, via Orabona 4, 70125 Bari, Italy

**Keywords:** Formyl peptide receptor 2, Compound MR-39, Organotypic hippocampal cultures, APP/PS1 double-transgenic mouse model, Alzheimer’s disease, NLRP3 inflammasome pathway

## Abstract

The major histopathological hallmarks of Alzheimer’s disease (AD) include β-amyloid (Aβ) plaques, neurofibrillary tangles, and neuronal loss. Aβ 1–42 (Aβ_1-42_) has been shown to induce neurotoxicity and secretion of proinflammatory mediators that potentiate neurotoxicity. Proinflammatory and neurotoxic activities of Aβ_1-42_ were shown to be mediated by interactions with several cell surface receptors, including the chemotactic G protein-coupled N-formyl peptide receptor 2 (FPR2). The present study investigated the impact of a new FPR2 agonist, MR-39, on the neuroinflammatory response in ex vivo and in vivo models of AD. To address this question, organotypic hippocampal cultures from wild-type (WT) and FPR2-deficient mice (knockout, KO, FPR2^−/−^) were treated with fibrillary Aβ_1-42_, and the effect of the new FPR2 agonist MR-39 on the release of pro- and anti-inflammatory cytokines was assessed. Similarly, APP/PS1 double-transgenic AD mice were treated for 20 weeks with MR-39, and immunohistological staining was performed to assess neuronal loss, gliosis, and Aβ load in the hippocampus and cortex. The data indicated that MR-39 was able to reduce the Aβ_1-42_-induced release of proinflammatory cytokines and to improve the release of anti-inflammatory cytokines in mouse hippocampal organotypic cultures. The observed effect was apparently related to the inhibition of the MyD88/TRAF6/NFкB signaling pathway and a decrease in NLRP3 inflammasome activation. Administration of MR-39 to APP/PS1 mice improved neuronal survival and decreased microglial cell density and plaque load.

These results suggest that FPR2 may be a promising target for alleviating the inflammatory process associated with AD and that MR-39 may be a useful therapeutic agent for AD.

## Introduction

Alzheimer’s disease (AD) is a chronic neurodegenerative disorder characterized by a gradual and progressive decline in cognitive functions. The neuropathological hallmarks of AD are the formation of intracellular neurofibrillary tangles from hyperphosphorylated tau protein and the aggregation of the amyloid-β peptide (Aβ) in extracellular plaques [1, 2]. The term Aβ is referred to a group of 37–43 amino acid peptides which are generated from the cleavage of the amyloid precursor protein. The Aβ_1-42_ peptide (42 amino acid residues) is one of the major Aβ forms in the brain and is closely linked to the development of AD [3]. Aβ_1-42_ can exert direct neurotoxic effects and stimulate the recruitment and activation of microglia and astrocytes to produce toxic inflammatory mediators, which ultimately lead to progressive neuronal death and synaptic loss [4, 5]. The role of chronic activation of microglia in AD is still unclear. Microglial cells are the major immune cells in the central nervous system (CNS) and are involved in the recognition of pathogens [6]. In AD, microglial activation may play a dual role. On the one hand, acute microglial activation has a protective function, leading to a decrease of Aβ accumulation by increasing its phagocytosis. On the other hand, chronic activation of microglia contributes to neurotoxicity by triggering proinflammatory cascades [7]. Similarly, chronic activation of astrocytes is a pathological hallmark of several neurological disorders and the extent of cognitive decline in AD has been correlated with the degree of astrogliosis [8]. Several studies have demonstrated that Aβ_1-42_ mediates proinflammatory and neurotoxic effects through the interactions with several cell surface receptors, including specific G protein-coupled receptors [9, 10].

The G protein-coupled Formyl Peptide Receptor 2 (FPR2) is a high-affinity binding partner of Aβ_1-42_. FPR2 belongs to the family of formyl peptide receptors, along with FPR1 and FPR3 [11] and it is expressed in immune cells, including glial cells [12]. In the CNS, FPR2 has been detected in the brainstem, spinal cord, thalamus/hypothalamus, cerebral neocortex, hippocampus, cerebellum, and striatum [13]. FPR2 is characterized by complex functional properties as it can be activated by chemically diverse ligands that stimulate different intracellular signalling pathways, depending on the chemical structure of the ligand and/or the cell type [14].

As mentioned above, FPR2 is a high-affinity binding partner of Aβ_1-42_. Upon binding to FPR2, Aβ_1-42_ is rapidly internalized into the cytoplasmic compartment of mononuclear phagocytes (monocytes and microglia). Transient FPR2 activation by Aβ stimulates rapid degradation of the peptide, whereas chronic stimulation contributes to the formation of fibrillar aggregates [15]. In addition, the interaction of Aβ_1-42_ with FPR2 is associated with the release of pro-inflammatory mediators [16, 17]. Moreover, FPR2 mediates the pro-inflammatory effects triggered by mitochondrial and bacterial N-formyl peptides, serum amyloid A, and prion protein PrP106-126 [18]. On the other hand, FPR2 mediates anti-inflammatory and pro-resolving effects, when activated by Annexin A1 (AnxA1) [19] and by the specialized pro-resolving mediators Lipoxin A4 (LXA4) [20], Resolvin D1 (RvD1) [21], and RvD3 [22]. For example, LXA4 and AnxA1 induce neutrophil apoptosis and macrophage efferocytosis [23]. The binding of AnxA1 to FPR2 has been associated with the modulation of both Aβ_1-42_ phagocytosis and the release of proinflammatory cytokines [24, 25].

These pieces of evidence suggest that FPR2 may play a complex role in the inflammatory process of AD. Zhang et al. recently reported that FPR2 deficiency can improve cognition and attenuate tau phosphorylation and astrogliosis in the streptozocin-induced mouse model of AD [26]. Consistently, Schröder et al. reported that blockade of FPR1 and FPR2 by the antagonist Boc2 in amyloid precursor protein/presenilin 1 (APP/PS1) transgenic mice ameliorates neuropathological deficits with a reduction in Aβ plaques in the hippocampus and an improvement in spatial memory performance [27]. On the other hand, administration of the pro-resolving mediator LXA4 in Tg2576 mice stimulates alternative activation of microglia with improved Aβ phagocytosis and clearance that alleviates synaptotoxicity and improves cognition [28]. These findings suggest that both the inhibition of FPR2 by an antagonist administration and the activation of FPR2 by a pro-resolving agonist may lead to beneficial effects on the hallmarks of AD, even though the involvement of different molecular mechanisms. Therefore, FPR2 has been proposed as a target for the development of a therapy for AD.

We recently identified the FPR2 agonist MR-39, which has neuroprotective and anti-inflammatory properties in LPS-stimulated primary rat microglial cells and is able to block LPS-induced cell death and proinflammatory cytokine production [29].

Based on these results and considering the potential complex role of FPR2 in the neuroinflammatory process in AD, we investigated the potential of MR-39 in alleviating the inflammatory response induced by Aβ, through the activation of pro-resolving pathways. In particular, we studied the impact of MR-39 on the inflammatory response ex vivo in organotypic hippocampal cultures (OHCs) from wild-type (WT) mice and FPR2-deficient mice (knockout, KO, FPR2^−/−^) treated with fibrillar amyloid-β_1-42_ (Aβ_1-42_) and then in vivo in APP/PS1 double-transgenic mice. In WT and KO FPR2^−/−^ organotypic hippocampal cultures, the effect of MR-39 on cell death, release of nitric oxide, and the levels of pro- and anti-inflammatory cytokines (IL-1β, TNF-α, IL-6, IL-4, and IL-10) were assessed under normal conditions and after treatment with fibrillar Aβ_1-42._ The effect of MR-39 was compared to that of the FPR2 antagonist WRW4. Next, to investigate the intracellular mechanisms of action of MR-39 in organotypic hippocampal cultures obtained from both WT and KO FPR2^−/−^ mice; we analyzed the intracellular pathways related to the inflammatory response, including MyD88/TRAF6/NF-кB and inflammasome 3 (NLRP3) signalling.

Finally, MR-39 was chronically administered to APP/PS1 mice to address the question of whether the activation of FPR2 has a beneficial effect on neuronal damage, inflammatory response, glial cell activation, and plaque density formation in the frontal cortex and hippocampus, which are the brain areas engaged in AD pathology.

## Materials and Methods

### Chemicals

Compound MR-39 ((*S*)-3-(4-cyanophenyl)-*N*-[[1-(3-chloro-4-fluorophenyl)cyclopropyl]methyl]-2-[3-(4-fluorophenyl)ureido]propanamide) was prepared as described previously in Stama et al. (2017) [29]. The FPR2 antagonist WRW4 was purchased from Alomone Labs, Israel. Aβ_1-42_ (Kaneka Eurogentec Inc, Belgium) was prepared according to the commonly accepted method described by Dahlgren et al. (2002) [30]. Briefly, Aβ_1-42_ was dissolved in HFIP (hexafluoroisopropanol, Sigma-Aldrich, St. Louis, MO, USA). Next, HFIP was removed under vacuum, and the peptide film was stored at − 20 °C. For the aggregation studies, the peptide was initially resuspended in DMSO (Sigma-Aldrich, St. Louis, MO, USA). For oligomeric conditions, culture media was added, and the peptide was incubated at 4 °C for 24 h. For fibrillar conditions, 10 mM HCl was added, and the peptide was incubated for 24 h at 37 °C [30, 31].

### Animals

KO FPR2^−/−^ were obtained from Dr. Lars-Ove Brandenburg of the Department of Anatomy and Cell Biology, RWTH Aachen University, Aachen, Germany. Briefly, KO FPR2^−/−^ mice on a C57BL/6 background were generated by mating single transgenic mice as described previously [32, 33]. Wild-type (WT) littermates on a C57BL/6 background were used as controls.

For ex vivo experiments, mice were maintained under standard conditions (room temperature of 23 °C, 12/12 h light/dark cycle, lights on at 06:00 am), with food and water available ad libitum. The experimental procedures were performed in accordance with the guidelines of the Committee for Laboratory Animal Welfare and Ethics of the Maj Institute of Pharmacology, Polish Academy of Sciences, Cracow, Poland.

The APP/PS1 double-transgenic mouse model used in this study (APPswe/PS1dE9-Line 85) coexpresses chimaeric mouse/human amyloid precursor protein (APP) 695 harboring the Swedish K670M/N671L mutation (Mo/HuAPPswe) and human presenilin 1 (PS1) with the exon-9 deletion (PS1dE9) under the control of the mouse prion protein promoter (Jankowsky et al., 2007). The mouse line was obtained from Jackson Laboratory (B6.Cg-Tg(APPswe,PSEN1dE9)85Dbo/J; stock no. 005864). WT littermates on a C57BL/6 background were used as controls. APP/PS1 mice were generated by mating single transgenic mice. WT mice obtained by mating were used as controls. The total number of mice used was as follows: WT *n* = 12, WT + MR39 *n* = 5, APP/PS1 *n* = 10, and APP/PS1 + MR39 *n* = 6. All animal experiments were approved by the Animal Care Committee of the University Hospital of Aachen and by the District Government in Recklinghausen, North Rhine-Westphalia, Germany (reference number 84–02.04. 2014. A399).

### Establishment of Hippocampal Organotypic Cultures

Six- to 7-day-old WT and KO FPR2^−/−^ mouse pups were used to prepare hippocampal organotypic cultures (OHCs) according to the method described by Stoppini et al. [34] with slight modifications. Briefly, the animals were decapitated, and the brain was quickly removed and placed in sterile ice-cold working buffer (96% HBSS, 3.5% glucose, 0.5% penicillin/streptomycin; all reagents were obtained from Gibco, UK). The isolated hippocampi were placed on Teflon disks and cut into 350 µm sections using a McIlwain tissue chopper. Then, the sections were transferred to ThinCerts™ TC inserts with 0.4 µm pore diameter membranes (Greiner Bio-One, Austria) in 6-well plates containing 1 ml of culture medium (50% DMEM + GlutaMax™-I, pH 7.4; 20.5% HBSS; 25% horse serum; 0.1 mg/ml glucose; 1% amphotericin B; 0.4% penicillin and streptomycin; 1% B-27 supplement; and HEPES (to maintain pH at 7.4); all reagents were obtained from Gibco, UK). The culture was grown in an incubator (37 °C) with 5% CO_2_ for 7 days (DIV 7). The cultures were initiated in regular medium containing 25% horse serum, which was then gradually (from DIV 4 to 7) decreased to serum-free medium (50% DMEM F-12, pH 7.4; 44% HBSS; 0.1 mg/ml glucose; 1% amphotericin B; 0.4% penicillin and streptomycin; 1% B-27; 1% N-2; and HEPES (to maintain pH at 7.4); all reagents were obtained from Gibco, UK). The medium was initially changed 24 h after the culture was established (half of the total volume, i.e., 0.5 ml) and then every 48 h (whole volume, i.e., 1 ml). On day 7 in vitro, the medium was changed to serum-free medium.

### OHC Treatments

OHCs obtained from WT and KO FPR2^−/−^mice were pretreated for 30 min with the FPR2 antagonist WRW4 (10 µM). Then, MR-39 (1 µM) was added for 1 h, and OHCs were stimulated for 24 h with fibrillary or oligomeric amyloid β (Aβ_1-42_; 10 μM). Based on the results of our preliminary experiments, we used fibrillar Aβ_1-42_ at a dose of 10 µM. Control (unstimulated) OHCs were treated with vehicle (phosphate-buffered saline (PBS))**.**

### Lactate Dehydrogenase Activity Assay

Lactate dehydrogenase (LDH) activity assay was used to measure the level of lactate dehydrogenase released to the culture medium as described previously [35]. The LDH level was measured 24 h after stimulation with fibrillar Aβ_1-42_ (10 μM) or oligomeric Aβ_1-42_ (10 μM) and/or MR-39 pretreatment. To quantify cell death, 50 µl of the supernatant collected from each well was incubated in 96-well plates with reagent mixture according to the supplier’s protocol (a cytotoxicity detection kit, Roche, Germany). The intensity of the red color formed in the colorimetric assay was measured at a wavelength of 490 nm (Infinite® 200 PRO plate reader, Tecan, Switzerland) and was proportional to LDH activity and the number of damaged/dead cells. The data were normalized to the activity of LDH released from the control samples (100%; vehicle-treated WT OHCs) and expressed as the percentage of the control ± SEM (standard error of the mean).

### Nitric Oxide Release Assay

The level of nitric oxide (NO) was measured by the Griess reaction in OHC culture media. According to the protocol, 24 h after stimulation with fibrillar Aβ_1-42_ (10 μM) or oligomeric Aβ_1-42_ (10 μM) and/or MR-39 pretreatment, 50 µl of the supernatant was mixed with an equal volume of Griess reagent (Griess A, 0,1% N-1-naphthylethylenediamine dihydrochloride and Griess B, 1% sulfanilamide in 5% phosphoric acid; Sigma-Aldrich) in a 96-well plate. The absorbance was measured at a wavelength of 540 nm using an Infinite® 200 PRO plate reader (Tecan) as described previously [36]. The data were normalized to the level of NO released from vehicle-treated cells (100%; vehicle-treated WT OHCs) and expressed as the percentage of the control ± SEM.

### Enzyme-Linked Immunosorbent Assays

Twenty-four hours after fibrillar Aβ_1-42_ (10 μM) treatment, OHC medium was collected to assess the levels of IL-1β, TNF-α, IL-6, IL-4 and IL-10. The protein levels of the cytokines (enzyme-linked immunosorbent assays (ELISA) kits for the detection of interleukin 1β (IL-1β), interleukin 4 (IL-4), interleukin 6 (IL-6), interleukin 10 (IL-10), and tumour necrosis factor α (TNF-α) were obtained from Cusabio, Houston, USA) were measured in the culture medium using commercially available enzyme-linked immunosorbent assay kits according to the manufacturers’ instructions. Additionally, 24 h after the treatment, OHCs were lysed with RIPA lysis buffer (Sigma-Aldrich, St. Louis, MO, USA) containing a protease inhibitor cocktail (Sigma-Aldrich, St. Louis, MO, USA), phosphatase inhibitor cocktail (Sigma-Aldrich, St. Louis, MO, USA), 1 mM sodium orthovanadate (Sigma-Aldrich, St. Louis, MO, USA), and 1 mM phenylmethanesulfonyl fluoride (Sigma-Aldrich, St. Louis, MO, USA). Protein content analysis of the samples was performed using a BCA protein assay kit (Sigma-Aldrich, St. Louis, MO, USA) according to the supplier’s instructions, and optical density was measured using an Infinite 200 Pro spectrophotometer (Tecan). The protein levels of NLRP3 ((NACHT, LRR, and PYD domains-containing protein 3 ELISA kit), NF-кB (nuclear factor-kappa B; both ELISA kits were from Cusabio Houston, USA), MyD88 (Myeloid differentiation primary response 88), CASP-1 (Caspase-1), PYCARD (Pyrin Domain-Containing Protein 3, ELISA kit for PYD and CARD Domain-Containing Protein), TRAF6 (TNF Receptor Associated Factor 6); all ELISA kits from ELK Biotechnology, Wuhan, China) and pNF-кB (NF-кB p65 (phospho) InstantOne ELISA kit, Thermo Fisher, Waltham, MA, USA) in the OHC lysates were measured using commercially available enzyme-linked immunosorbent assay kits according to the manufacturers’ instructions. The detection limits were as follows: IL-1β < 7.8 pg/mL, IL-4 < 0.39 pg/mL, IL-6 < 0.39 pg/mL, IL-10 < 0.78 pg/mL, TNF-α < 3.9 pg/mL, MyD88 < 0.061 ng/mL, CASP1 < 13.5 pg/mL, PYCARD < 3 pg/mL, TRAF6 < 0.53 ng/mL, NLRP3 < 1.56 pg/mL, NF-κB < 0.078 ng/mL, and NF-κB p65 (phospho) not applicable. Inter-assay precision of all ELISA kits was CV% < 10%. Intra-assay precision of all ELISA kits was CV% < 8%.

### Drug Treatment for *In Vivo* Experiments

To study the protective effects of FPR2 activation in an AD mouse model, 8-week-old APP/PS1 double-transgenic or WT mice were intraperitoneally (i.p.) injected with MR-39 (10 mg/kg) twice a week for a period of 20 weeks. The subsequent assays were performed by experimenters blinded to the treatment groups. The mice were sacrificed at an age of 29 weeks.

### Immunohistochemistry

For immunohistochemistry, brain sections were rehydrated, and the antigens were unmasked if necessary using Tris/EDTA buffer (pH 9.0) or citrate (pH 6.0) heating as described previously (Bihler et al., 2017). For GFAP staining, the sections were washed in PBS and incubated overnight at 4 °C with an anti-GFAP antibody (1:75,000; RPCA-GFAP, EnCor, Gainesville, FL, USA) diluted in blocking solution (containing serum of the species corresponding to the source of a secondary antibody). On the next day, the slides were incubated with 0.3% H_2_O_2_ in PBS for 30 min and then with biotinylated secondary antibodies (1:50; BA-1000; Biozol, Eching, Germany) for 1 h. After a washing step, the slides were incubated with peroxidase-conjugated avidin–biotin complex (ABC kit; Vector Laboratories, Peterborough, UK) and subsequently treated with 3,3′-diaminobenzidine (DAKO, Hamburg, Germany) as a peroxidase substrate. Finally, the slides were counterstained with hematoxylin and covered with DePeX (Serva, Heidelberg, Germany). For immunofluorescence staining, the slides were incubated with rabbit anti-beta-amyloid 1–42 (1:150; AB5078P, Merck Millipore, Darmstadt, Germany), mouse anti-Iba-1 (1:1000; MABN92, Merck Millipore, Darmstadt, Germany) or rabbit anti-NeuN (1:250; ab177487, Abcam, Cambridge, United Kingdom) antibodies and subsequently incubated with anti-rabbit IgG Alexa Fluor 594 or anti-mouse IgG Alexa Fluor 488 secondary antibodies (1:250; A11012 or A11001, Thermo Fisher Scientific, Dreieich, Germany). Cell nuclei were visualized using bisbenzimide (1:10.000 in PBS), and the section were mounted in Immuno-Mount (Thermo Fisher Scientific).

### Quantification of Immunoreactive Cells

Stained and processed sections were digitized using a Keyence Analysis software imaging system (a Keyence BZ-9000 microscope; Keyence, Neu-Isenburg, Germany). Hippocampal formation and somatosensory and motor cortices were defined as regions of interest (ROIs). In general, three randomly selected slides were processed and evaluated per staining type and experimental animal.

Various strategies were used for (semi-) quantitative evaluation of staining intensity as reported previously [27]. For Aβ_1-42_ plaques stained with anti-beta-amyloid 1–42, four different size categories (> 75–125 µm^2^, 125–250 µm^2^, 250–500 µm^2^, and > 500 µm^2^) were defined, and individual areas per plaque were quantified using a modified version of the “Analyze particles macro” of ImageJ. Microglial reactivity around the plaques was analyzed in anti-IBA1-stained sections in a circular area around the plaque center (diameter of 50 µm). The 50 µm diameter was selected due to spatial proximity of Aβ_1-42_ plaques to prevent overlapping. The extent of microglial activation around the plaques was assigned an IBA1^+^ area minus the plaque area in µm^2^, and the values were grouped according to the size of the plaques based on the categories described above. To quantify neuronal cell density, the layer V of the motor and somatosensory cortex was delineated in NeuN-stained sections, and NeuN^+^ cells were manually counted using ImageJ. Fluorescence intensity of the signal of anti-NeuN staining was determined, expressed as fluorescence intensity in % per hippocampal area, and used to estimate neuronal cell density in the dentate gyrus.

### Statistical Analysis

#### Ex Vivo* Experiments*

Statistical analysis was performed using the Statistica 10.0 software (Stat Soft, Tulsa, USA). All biochemical experiments were carried out under the same conditions for all samples, regardless of the type of the treatment. The results presented in this study were derived from three independent KO FPR2^−/−^ or WT OHCs, and “n” for each culture was 2–5. All data were obtained in independent experiments and are presented as the mean ± SD (standard deviation). All groups were compared using factorial analysis of variance (ANOVA) to determine the effects of the factors followed, when appropriate, by Duncan’s post hoc test. A *p* value less than 0.05 was considered to be statistically significant. All graphs were prepared using GraphPad Prism 5.

## Results

### Aβ_1-42_ Increases Cell Death in OHCs Obtained from the Offspring of WT and KO FPR2^−/−^ Mice

**Fig. 1:**
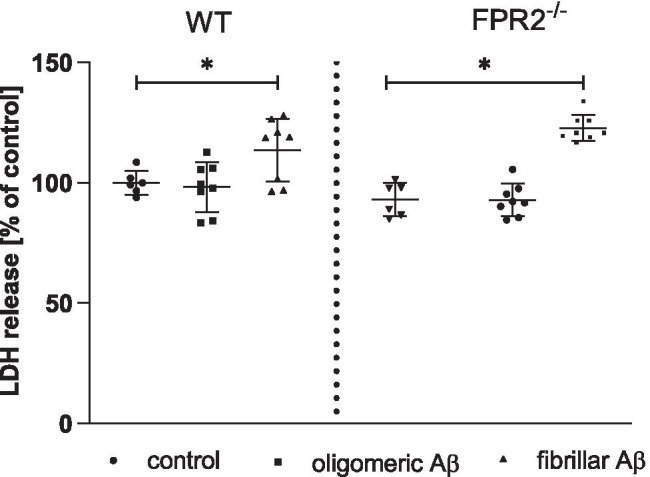
The influence of oligomeric and fibrillar types of β-amyloid on cell death in OHCs obtained from the offspring of WT and KO FPR2 mice. OHCs were stimulated for 24 h with fibrillar or oligomeric amyloid β (Aβ_1-42_; 10 μM). Control cultures were treated with the appropriate vehicle. The effect of β-amyloid (oligomeric Aβ_1-42_ and fibrillar Aβ_1-42_) on cell death (LDH) was measured. The data are presented as the mean ± SD, percentage of the control values (vehicle-treated WT OHCs) obtained in independent experiments. The results were statistically evaluated using a two-way analysis of variance (ANOVA) with the Duncan post hoc test to assess the differences between the treatment groups. Significant differences are indicated by ∗ *p* < 0.05

The impact of two types of β-amyloid (oligomeric Aβ_1-42_ and fibrillar Aβ_1-42_) on lactate dehydrogenase release (LDH) was examined in hippocampal organotypic cultures in the initial experiments. The LDH assay is a well-accepted paradigm for quantitative assessment of cell death after damage to the plasma membrane, which results in an increase in LDH efflux from injured cells. As shown in Fig. 1, fibrillar Aβ_1-42_ increased cell death in OHCs obtained from both WT (*p* = 0.002427) and KO FPR2^−/−^ (*p* = 0.005377) mice. In contrast, oligomeric Aβ_1-42_ had no effect on LDH release. Thus, we used fibrillar Aβ_1-42_ at a dose of 10 µM in the next set of experiments.

### MR-39 Treatment Diminishes Aβ_1-42_-Induced LDH Release in OHCs from the Offspring of WT but not KO FPR2^−/−^ Mice

**Fig. 2:**
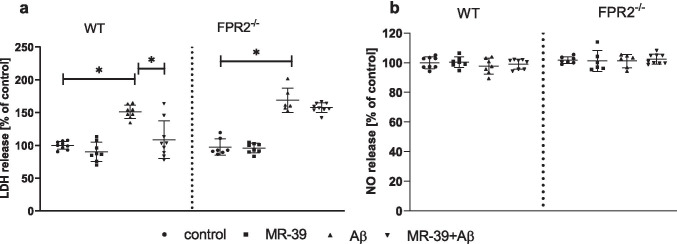
MR-39 treatment diminishes Aβ_1-42_-induced LDH release in OHCs from the offspring of WT but not KO FPR2 mice. OHCs were stimulated for 24 h with fibrillar amyloid β (Aβ_1-42_; 10 μM). Control cultures were treated with the appropriate vehicle. NO release was measured by the Griess reaction. The data are presented as the mean ± SD, percentage of the control values (vehicle-treated WT OHCs) obtained in independent experiments. The results were statistically evaluated using a two-way analysis of variance (ANOVA) with the Duncan post hoc test to assess the differences between the treatment groups. Significant differences are indicated by ∗ *p* < 0.05. LDH, lactate dehydrogenase; NO, nitric oxide

The impact of the FPR2 agonist MR-39 on cell death induced by Aβ_1-42_ treatment was evaluated. We confirmed the harmful effect of Aβ_1-42_ (10 μM) corresponding to an increase in LDH release (Fig. 2a) in OHCs obtained from both WT (*p* = 0.000481) and KO FPR2^−/−^ (*p* = 0.002257) mice. Interestingly, the pro-resolving properties of MR-39 (1 μM) were detected only in WT (*p* = 0.001056; Fig. 2a) cultures. Then, we used an assay based on the Griess reaction to assess the impact of fibrillar Aβ_1-42_ and MR-39 on NO secretion. As shown in Fig. 2b, fibrillar Aβ_1-42_ or MR-39 did not influence NO release in cultures obtained from WT and KO FPR2^−/−^ mice.

### MR-39 Treatment Diminishes Proinflammatory Cytokine Release Evoked by Aβ_1-42_ Treatment in OHCs from the Offspring of WT but not KO FPR2^−/−^ Mice

The levels of IL-1β, TNF-α, and IL-6 were assessed in cultures prepared from both WT and KO mice stimulated by fibrillar Aβ_1-42_ to evaluate the effect of MR-39 on the production of proinflammatory cytokines. Furthermore, OHCs were pretreated with MR-39 alone and with the selective FPR2 antagonist WRW4 to determine whether the effect of fibrillar Aβ_1-42_ was mediated by interaction with FPR2.

**Fig. 3:**
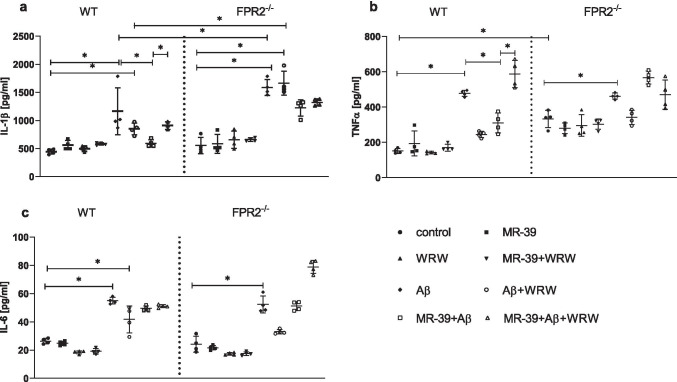
MR-39 treatment diminishes proinflammatory cytokine release evoked by Aβ_1-42_ treatment in OHCs from the offspring of WT but not KO FPR2 mice*.* OHCs were pretreated for 30 min with the FPR2 antagonist WRW4 (10 µM). Then, MR-39 (1 µM) was added for 1 h, and OHCs were stimulated for 24 h with fibrillar amyloid β (Aβ_1-42_; 10 μM). Control cultures were treated with the appropriate vehicle. The results are expressed as the mean ± SD. The data are from independent experiments. The results were statistically evaluated using factorial analysis of variance (ANOVA) with Duncan’s post hoc test to assess the differences between the treatment groups. Significant differences are indicated by ∗ *p* < 0.05. IL, interleukin; TNF-α, tumour necrosis factor α

Stimulation with fibrillar Aβ_1-42_ (10 μM) significantly increased the levels of IL-1β in the medium of WT (*p* < 0.0001) and KO (*p* < 0.0001) cultures. However, this effect was significantly more pronounced in KO (*p* = 0.000144) cultures, as shown in Fig. 3a. Under basal conditions, MR-39 (1 μM), WRW4 (10 μM), or MR-39 + WRW4 had no effect on IL-1β levels in WT and KO cultures (Fig. 3a). Treatment with Aβ_1-42_ (10 μM) and WRW4 (10 μM) induced a significant upregulation in IL-1β release in both, WT (*p* = 0.001920) and KO cultures (*p* < 0.0001). Importantly, the treatment with MR-39 (1 μM) decreased (*p* = 0.002425) the levels of IL-1β only in WT hippocampal cultures stimulated with fibrillar Aβ_1-42_ (10 μM), and pretreatment with the antagonist WRW4 blocked this beneficial effect of MR-39 on the secretion of IL-1β (*p* = 0.007073; Fig. 3a).

Under basal conditions, the level of TNF-α in OHCs obtained from KO mice was higher than that in hippocampal cultures obtained from WT mice (*p* < 0.0001; Fig. 3b). Fibrillar Aβ_1-42_ (10 μM) induced a significant upregulation of TNF-α production in WT (*p* < 0.0001) and KO (*p* = 0.00055) cultures (Fig. 3b). This effect was abolished after the treatment with MR-39 (1 μM) (*p* = 0.000194) only in OHCs from WT mice. The beneficial impact of MR-39 on hippocampal WT cultures was blocked by WRW4 (*p* < 0.0001; Fig. 3b).

Treatment with fibrillar Aβ_1-42_ (10 μM) upregulated IL-6 production in both WT (*p* < 0.0001) and KO cultures (*p* < 0.0001). Moreover, treatment with Aβ_1-42_ (10 μM) and WRW4 (10 μM) also lead to increased production of IL-6 but only in WT cultures (*p* < 0.0001). No other differences between the treatment groups of WT and KO cultures were detected (Fig. 3c).

### MR-39 Treatment Increases the Release of Anti-Inflammatory Cytokines in OHCs Obtained from the Offspring of WT but not KO FPR2^−/−^ Mice

**Fig. 4:**
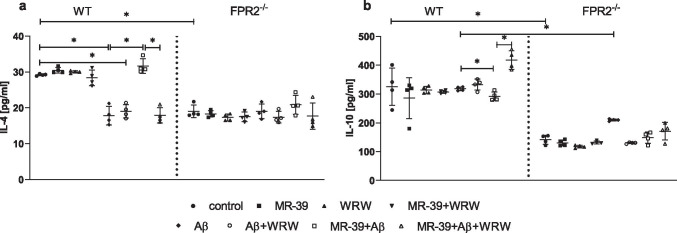
MR-39 treatment increases the release of anti-inflammatory cytokines in OHCs obtained from the offspring of WT but not KO FPR2 mice. OHCs were pretreated for 30 min with the FPR2 antagonist WRW4 (10 µM). Then, MR-39 (1 µM) was added for 1 h, and OHCs were stimulated for 24 h with fibrillar amyloid β (Aβ_1-42_; 10 μM). Control cultures were treated with the appropriate vehicle. The results are expressed as the mean ± SD. The data are from independent experiments. The results were statistically evaluated using factorial analysis of variance (ANOVA) with Duncan’s post hoc test to assess the differences between the treatment groups. Significant differences are indicated by ∗ *p* < 0.05. IL – interleukin

Then, the impact of fibrillar Aβ_1-42_, compounds MR-39 and/or WRW4 on the release of anti-inflammatory cytokines was evaluated in OHCs from both WT and KO mice. Interestingly, under basal conditions, the levels of IL-4 (*p* = 0.000119) and IL-10 (*p* < 0.0001) in KO cultures were significantly lower than those in similar cultures obtained from WT mice (Fig. 4a, b). Furthermore, in WT cultures, stimulation with fibrillar Aβ_1-42_ (10 μM), as well as with combination (Aβ_1-42_ + WRW4), significantly downregulated the production of IL-4 (*p* < 0.0001; *p* < 0.0001, respectively), and this effect was reversed by MR-39 (*p* < 0.0001). WRW4 inhibited the beneficial effect of MR-39 (*p* < 0.0001) (Fig. 4a). These effects were not observed in KO cultures.

Treatment with fibrillar Aβ_1-42_ (10 μM) had no effect on IL-10 production in WT cultures (Fig. 4b) and decreased the level of IL-10 in KO cultures compared with that in WT OHCs (*p* < 0.0001). On the other hand, treatment with MR-39 decreased the release of IL-10 in WT cultures treated with fibrillar Aβ_1-42_ (*p* < 0.0001), and a WRW4 antagonist reversed this unexpected effect of MR-39 (*p* < 0.0001).

### MR-39 Treatment Decreases the Synthesis of MyD88 and NF-kB Induced by Aβ_1-42_ in OHCs Obtained from the Offspring of WT but not KO FPR2^−/−^ Mice

The MyD88/TRAF6/NFkB pathway is one of the crucial signalling pathways activated by fibrillar Aβ_1-42_. Activation of the MyD88/TRAF6 pathway results in the activation of transcription factors, including NF-κB, and consequent release of some proinflammatory cytokines. Thus, the influence of fibrillar Aβ_1-42_ and/or MR-39 on the protein levels of MyD88 and TRAF6 was examined.

**Fig. 5:**
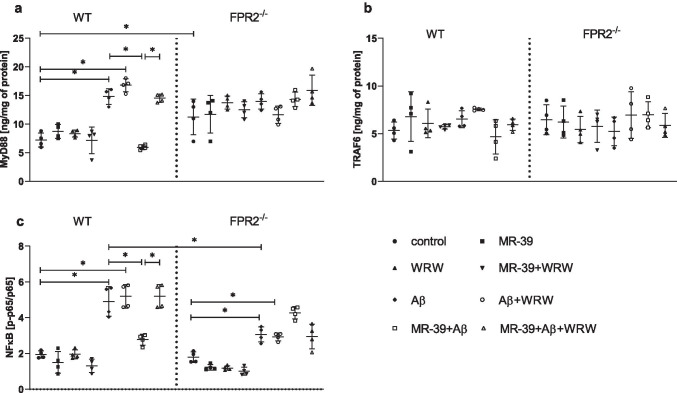
MR-39 treatment decreases the synthesis of MyD88 and NF-kB induced by Aβ_1-42_ administration in OHCs obtained from the offspring of WT but not KO FPR2 mice. OHCs were pretreated for 30 min with the FPR2 antagonist WRW4 (10 µM). Then, MR-39 (1 µM) was added for 1 h, and OHCs were stimulated for 24 h with fibrillar amyloid β (Aβ_1-42_; 10 μM). Control cultures were treated with the appropriate vehicle. The results are expressed as the mean ± SD. The data are from independent experiments. The results were statistically evaluated using factorial analysis of variance (ANOVA) with Duncan’s post hoc test to assess the differences between the treatment groups. Significant differences are indicated by ∗ *p* < 0.05. TRAF6, TNF receptor-associated factor 6; MyD88. myeloid differentiation primary response 88; NF-kB, nuclear factor-κB

The level of MyD88 in cultures obtained from KO mice was higher than that in WT hippocampal cultures (*p* = 0.03242; Fig. 5a). Fibrillar Aβ_1-42_ induced a significant upregulation of MyD88 levels only in WT (*p* < 0.000384) OHCs. Treatment with Aβ_1-42_ and WRW4 (*p* < 0.000384) leads to similar results. Importantly, MR-39 (1 μM) antagonized an increase in MyD88 levels evoked by fibrillar Aβ_1-42_ (*p* < 0.0001), and this effect was blocked by WRW4 (*p* < 0.0001) (Fig. 5a). This effect was not detected in the KO cultures. The tested factors did not influence the level of the TRAF6 protein in the hippocampal cultures in the studied groups (Fig. 5b).

MyD88 activation leads to the activation of the NF-кB pathway and expression of proinflammatory genes (especially IL-1β and TNF-α); thus, the effect of fibrillar Aβ_1-42_ and/or MR-39 and WRW4 on the phosphorylation level of p65 NF-κB was examined. As shown in Fig. 5c, the phospho-p65/total p65 ratio was similar in WT and KO cultures under basal conditions. On the other hand, an increase in the phospho-p65/total p65 ratio was observed after stimulation of WT (*p* < 0.0001) and KO cultures (*p* = 0.03499) with fibrillar Aβ_1-42_ (10 μM) (Fig. 5c) and when we used the Aβ_1-42_ and WRW4 (*p* < 0.0001; *p* = 0.031937, respectively). Moreover, upregulation of the phospho-p65/total p65 ratio was attenuated by MR-39 (*p* < 0.000105) treatment only in the WT cultures, and pretreatment with WRW4 blocked this effect (*p* < 0.00011) (Fig. 5c).

### MR-39 Treatment Diminishes the Synthesis of NLRP3, Caspase-1, and ASC Evoked by Aβ1-42 Administration in OHCs Obtained from the Offspring of WT but not KO FPR2^−/−^ Mice

Recent studies indicated that the NLRP3 inflammasome is an important platform involved in the secretion of proinflammatory cytokines, mainly IL-1β. Moreover, the MyD88/TRAF6/NFkB signalling pathway is involved in the priming of the activation of NLRP3 inflammasome in AD mouse models. Therefore, we estimated the impact of Aβ_1-42_ and/or MR-39 and WRW4 on the protein levels of the components of NLRP3 inflammasome, such as NLRP3, caspase-1, and ASC.

**Fig. 6:**
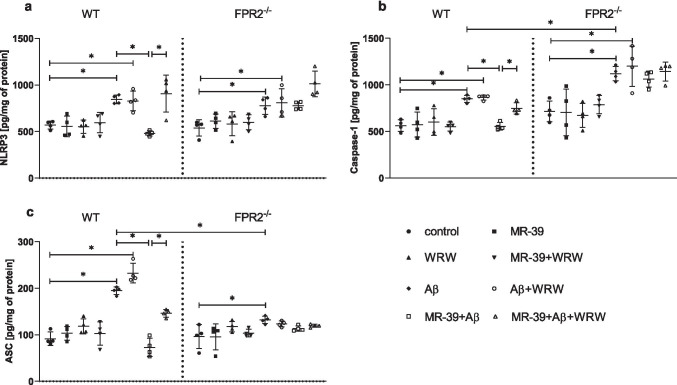
MR-39 treatment diminishes the synthesis of NLRP3, caspase-1 and ASC evoked by Aβ_1-42_ administration in OHCs obtained from the offspring of WT but not KO FPR2 mice. OHCs were pretreated for 30 min with the FPR2 antagonist WRW4 (10 µM). Then, MR-39 (1 µM) was added for 1 h, and OHCs were stimulated for 24 h with fibrillar amyloid β (Aβ_1-42_; 10 μM). Control cultures were treated with the appropriate vehicle. The results are expressed as the mean ± SD. The data are from independent experiments. The results were statistically evaluated using factorial analysis of variance (ANOVA) with Duncan’s post hoc test to assess the differences between the treatment groups. Significant differences are indicated by ∗ *p* < 0.05. ASC, apoptosis-associated speck-like protein containing a caspase recruitment domain; NLRP3, Nod-like receptor pyrin-containing 3 subunit

The levels of all proteins under basal conditions were similar in the WT and KO groups. Stimulation with Aβ_1-42_ (10 μM) increased the levels of NLRP3 and caspase-1 in both WT (*p* = 0.04606; *p* = 0.02243, respectively) and KO (*p* = 0.03655; *p* = 0.001472, respectively) OHCs (Fig. 6a, b). Similar effect was observed after treatment with Aβ_1-42_ and WRW4, which led to increase in the NLRP3 and Caspase-1 levels in both WT (*p* = 0.031381; *p* = 0.0207988, respectively) and KO hippocampal cultures (*p* = 0,023,034; *p* < 0.0001, respectively). Importantly, the upregulation of the level of caspase-1 by Aβ_1-42_ in KO cultures was significantly stronger than that in WT cultures (*p* = 0.02234) (Fig. 6a).

In the case of ASCs, upregulation after Aβ_1-42_ stimulation was observed in both WT (*p* < 0.0001) and KO cultures (*p* = 0.02675; Fig. 6c); however, this upregulation was weaker in KO cultures than that in OHCs obtained from WT mice (*p* < 0.0001). Importantly, MR-39 treatment effectively diminished an increase in the protein levels of the NLRP3 (*p* = 0.01319), caspase-1 (*p* = 0.013206), and ASC (*p* < 0.0001) subunits of inflammasome evoked by Aβ_1-42_ only in WT hippocampal cultures (Fig. 6a, b ,c). Moreover, pretreatment with the antagonist WRW4 blocked this beneficial effect of MR-39 (*p* = 0.01366; *p* = 0.02721; *p* < 0.0001, respectively).

### MR-39 Improves Neuronal Survival in Double-Transgenic APP/PS1 Mice

**Fig. 7:**
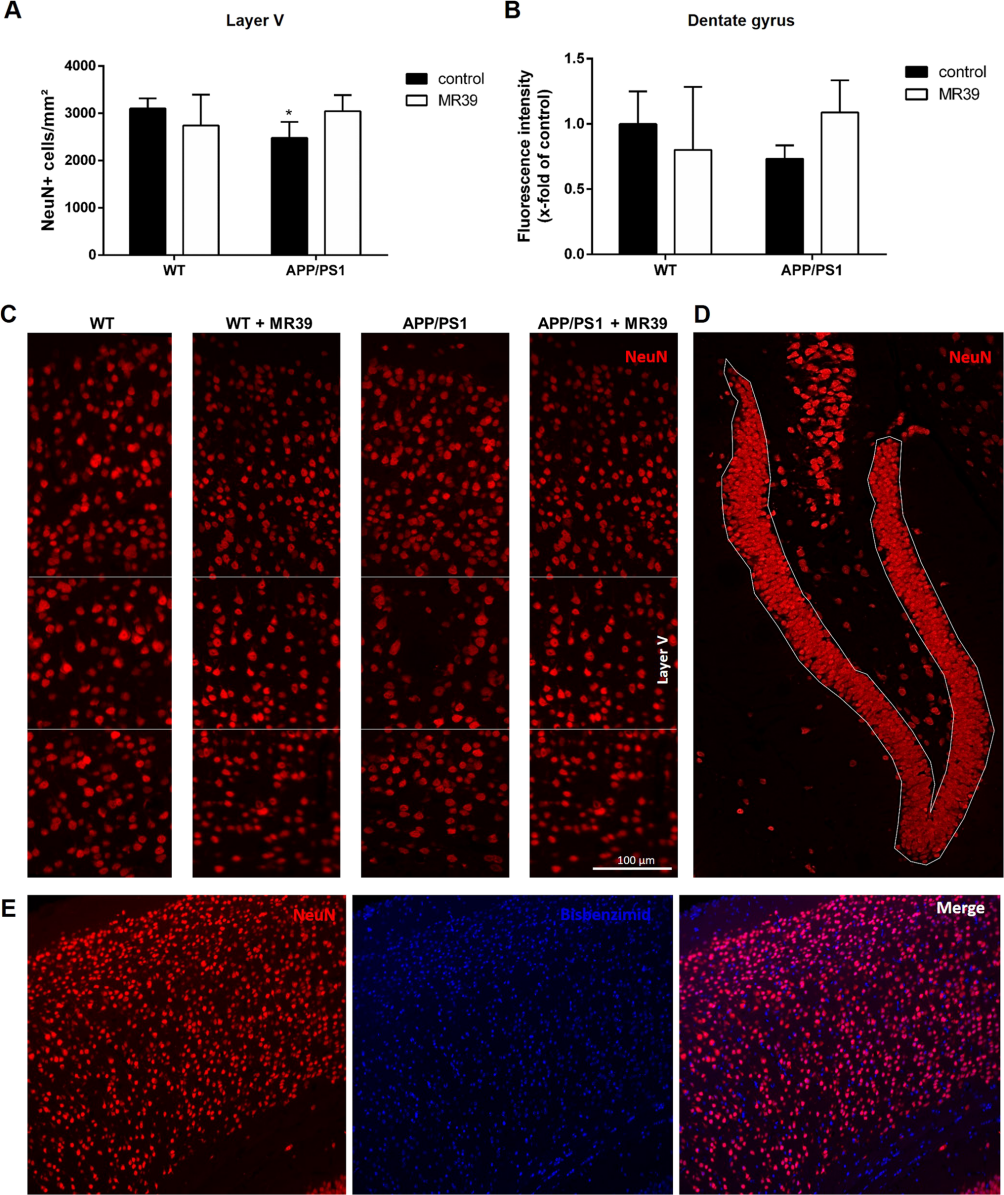
MR-39 treatment ameliorates neuronal loss in APP/PS1 double-transgenic mice. (**A**) Coronal brain sections of 29-week-old APP/PS1 double-transgenic or WT mice with or without MR-39 treatment were stained with anti-NeuN antibodies to label neuronal cells. Quantification of NeuN staining of (**A**) the layer V (cell density/mm^2^) or (**B**) dentate gyrus of the hippocampus (fluorescence intensity, %). Representative images of the cortex (**C**) and hippocampus (**D**) are shown. Representative co-staining of NeuN with Bisbenzimid (merge) shows the neuronal nucleus specifity of the antibody in the cortex (**E**). Statistical significance was determined using the Kruskal–Wallis test followed by Dunn’s post hoc test (**A**/**B**). Scale bar, **C** and **D**: 100 µm. The number of animals was as follows: WT *n* = 12, WT + MR39 *n* = 5, APP/PS1 *n* = 10, and APP/PS1 + MR39 *n* = 6). The data are presented as the mean ± SD; *n* ≥ 5; *p < 0.05

APP/PS1 double-transgenic and WT mice were used to test the effect of i.p. injection of MR39 to assess possible functional effect of MR-39 in AD. MR-39 was administered for a period of 20 weeks, and the density of neuronal cells was subsequently tested using NeuN as a marker for mature neurons in the layer V of the cortex and in the dentate gyrus of the hippocampus (Fig. 7). Similar to previous observations [27], mean neuronal densities were significantly decreased in the layer V of the somatosensory cortex and tended to decrease in the dentate gyrus in APP/PS1 double-transgenic mice compared to those in WT mice (Fig. 7a, b; Kruskal–Wallis test followed by Dunn test, *p* < 0.05 and *p* > 0.05). Treatment of the animals with MR39 tended to increase the NeuN density, although the differences were not significant (Fig. 7a, b; both *p* > 0.05).

### MR-39 Treatment Reduces the Number of Microglial Cells but not Astrocytes in APP/PS1 Double-Transgenic Mice

**Fig. 8:**
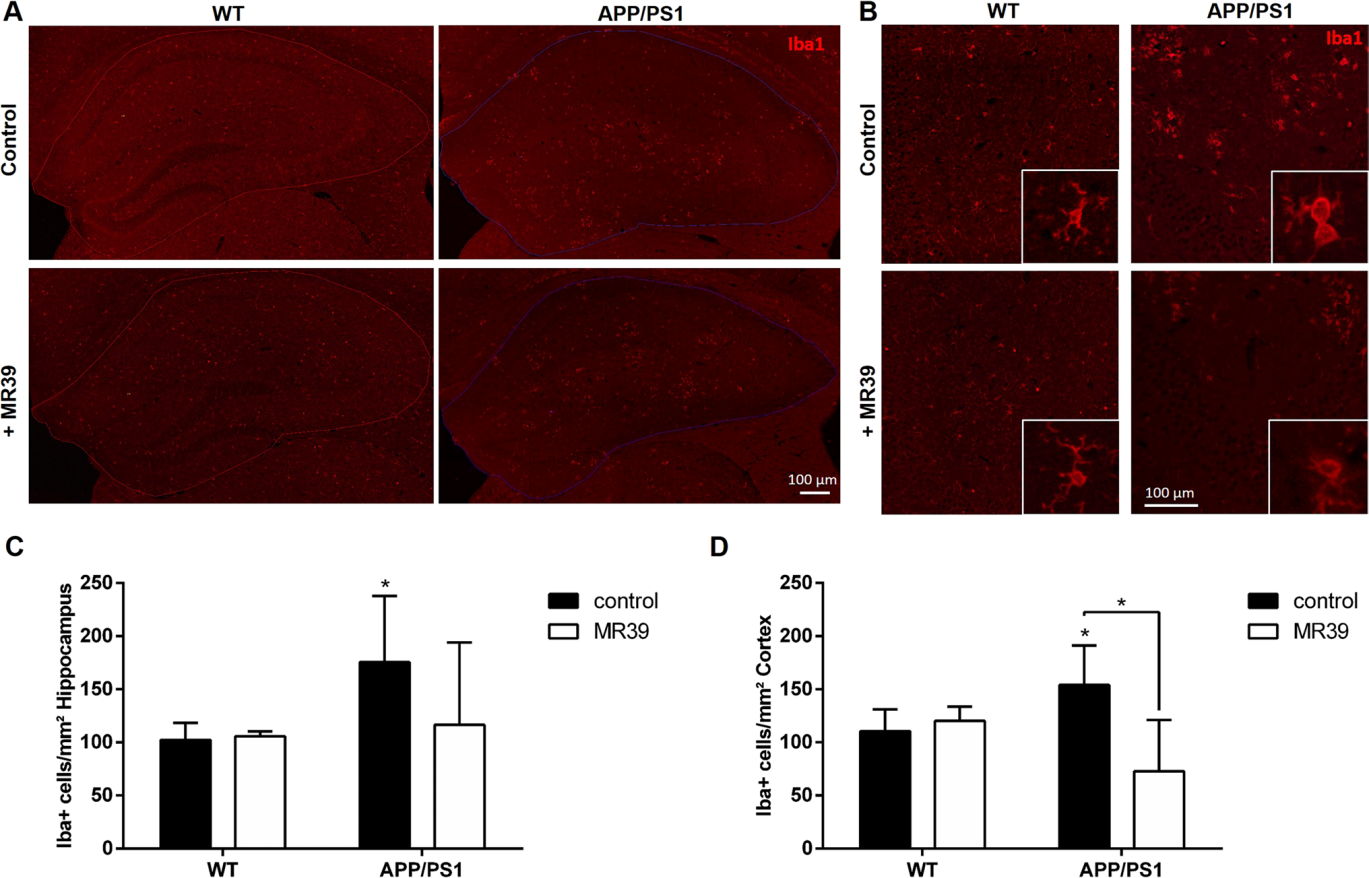
MR39 treatment ameliorates microglial cell reactivity in APP/PS1 double-transgenic mice. Coronal brain sections of 29-week-old APP/PS1 double-transgenic or WT mice with or without MR-39 treatment were stained with anti-IBA-1 as a marker for microglial cells. (**A**) Representative images of the entire hippocampus and (**B**) a higher magnification image of the dentate gyrus. Quantification of IBA-1 staining intensities within (**C**) the hippocampus or (**D**) cortex (cell density/mm^2^). Statistical significance was determined using two-way ANOVA with Tukey’s test (**C** and **D**). Scale bar, C: 100 µm. The data are presented as the mean ± SD; *n* ≥ 5; *p < 0.05

**Fig. 9:**
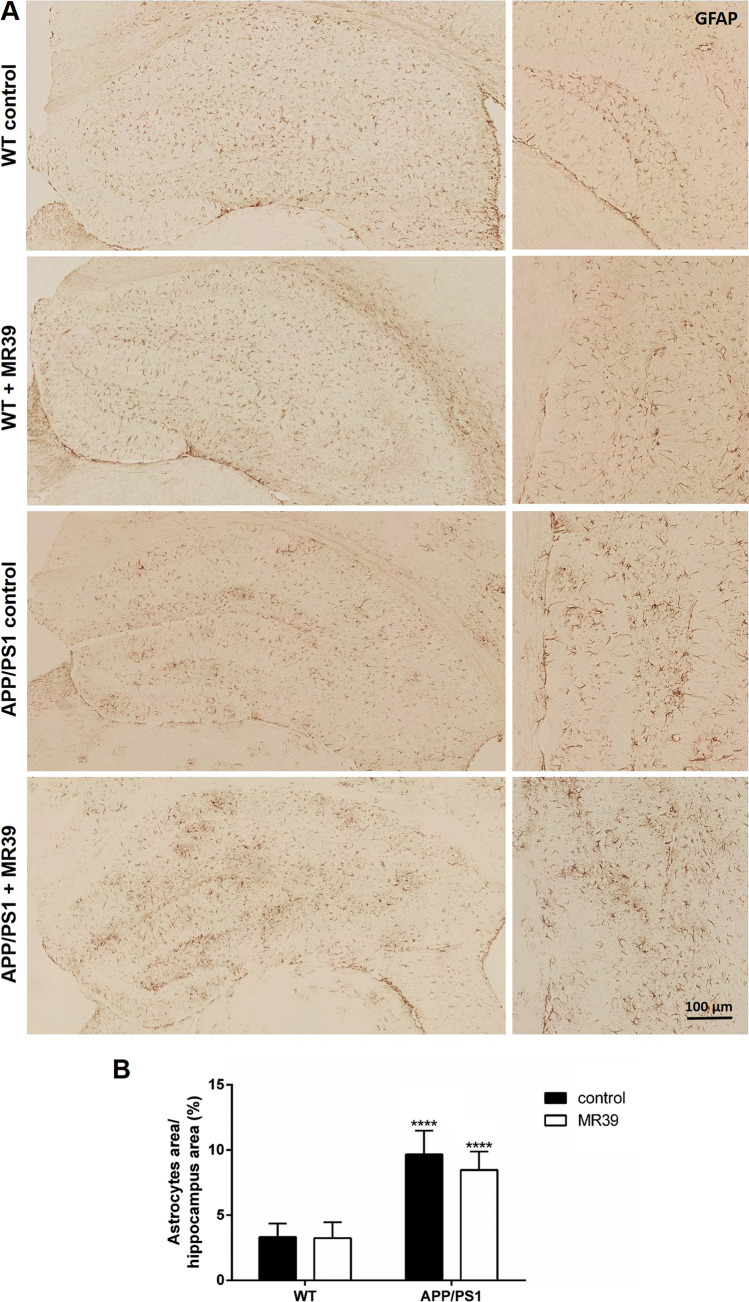
MR-39 does not affect astrocytes in APP/PS1 mice. (**A**) Representative anti-GFAP staining of the entire hippocampus as well as a higher magnification of the dentate gyrus region of WT, WT + MR39, APP/PS1, and APP/PS1 + MR39 mice. (**B**) An increase in GFAP-positive staining (cells/mm^2^) in the hippocampus of WT control and APP/PS1 control mice. Scale bar, A: 100 µm. The data are presented as the mean values of each group with SD. *n* ≥ 5; Two-way ANOVA with Turkey’s test; ****p < 0.0001

Next, we aimed to determine whether an increase in NeuN density in MR39-treated APP/PS1 double-transgenic mice was paralleled by less severe activation of microglia. As shown in Fig. 8, a significant increase in the numbers of microglial cells was observed in the hippocampus and cortex of APP/PS1 double-transgenic mice versus those in WT mice (Fig. 8c, d; two-way ANOVA with Tukey’s test, both *p* < 0.05). This increase in the numbers of microglial cells in the cortex was significantly ameliorated by MR-39 treatment, while no significant differences were detected in the hippocampus (cortex: 140.3 ± 18.7 vs. 72.9 ± 21.6 cells/mm^2^). Furthermore, astrocyte reactivity was analyzed using anti-GFAP-stained sections. A significant increase in the numbers of astrocytes was detected in the hippocampus of APP/PS1 double-transgenic mice versus that in WT mice (Fig. 9b; two-way ANOVA with Tukey’s test, *p* < 0.0001; hippocampus WT 3.3 ± 0.2 vs. APP/PS1 9.7 ± 0.4 astrocyte area/hippocampus area). Unlike the effects detected in microglial cells, astrocyte reactivity was not ameliorated by MR-39 treatment in APP/PS1 double-transgenic mice.

### MR-39 Treatment Reduces Plaque Load in APP/PS1 Double-Transgenic Mice

**Fig. 10:**
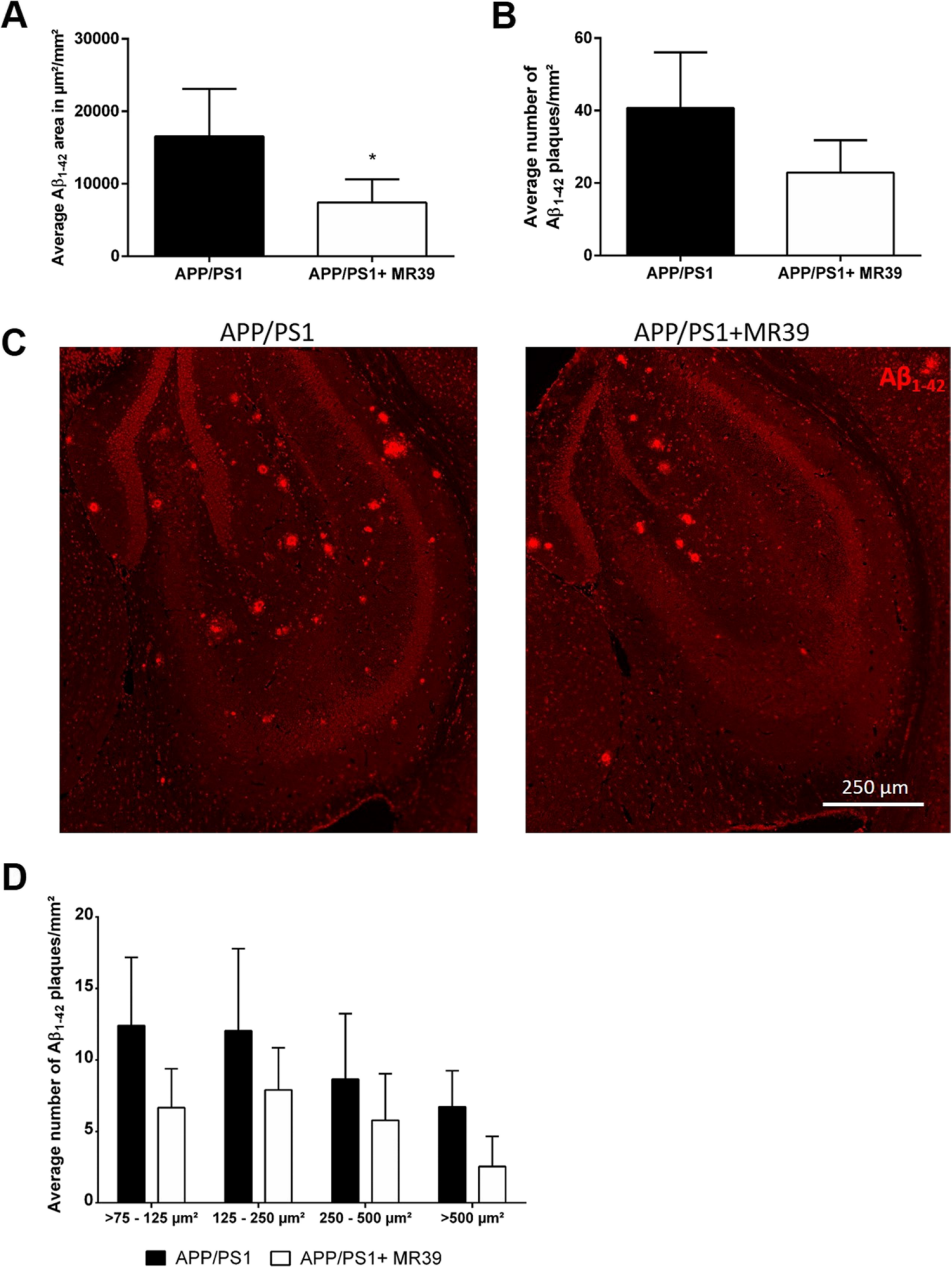
MR-39 treatment reduces the plaque load of Aβ in APP/PS1 double-transgenic mice. Coronal brain sections of 29-week-old APP/PS1 double-transgenic mice with or without MR39 treatment were stained with anti-beta-amyloid 1–42 and used for the determination of plaque load. (**A**) Analysis of the Aβ plaque area or (**B**) average number of Aβ plaques (number/mm^2^) in the hippocampus. (**C**) Representative images of hippocampal plaque analysis demonstrating the evaluation process using ImageJ. Scale bar: 250 μm. (**D**) The average number of plaques/mm^2^ grouped into four categories with various sizes (< 125 μm^2^, 125–250 μm^2^, 250–500 μm^2^, and > 500 μm^2^). Statistical significance was determined using the Mann–Whitney *U* test (**A** and **B**) or two-way ANOVA with Tukey’s test (**D**). The data are presented as the mean ± SD; *n* ≥ 5; *p < 0.05

The results indicated that microglia rather than astrocytes were modulated by MR-39 treatment; thus, the effects of Aβ_1-42_ were investigated in microglial cells. Aggregation of Aβ_1-42_ peptides to solid plaques is the typical hallmark of AD. Therefore, we analyzed the hippocampal plaque load. As shown in Fig. 10a, the total hippocampal plaque area was significantly reduced in MR39-treated mice compared to that in vehicle-treated APP/S1 double-transgenic mice (Fig. 10a; Mann–Whitney *U* test, *p* = 0.0318; 16,550 ± 2921 vs. 7405 ± 1437 plaque area/mm^2^). The average number of plaques per area was also reduced, although the reduction was not significant (Fig. 10b; Mann–Whitney *U* test). The numbers of small (> 75–125 µm^2^), medium (125–250 µm^2^), large (250–500 µm^2^), and very large (> 500 µm^2^) plaques were quantified to determine which plaque types (small versus large) were preferentially reduced by MR 39 treatment. As shown in Fig. 10d (two-way ANOVA with Tukey’s test), all plaque sizes presented a reduction in size; however, the numbers of the plaques were not significantly reduced in MR39-treated APP/PS1 double-transgenic mice.

## Discussion

Recently data suggest that FPR2 may play a complex role in the inflammatory process in AD. Indeed FPR2 is a high-affinity binding partner of Aβ_1-42_ that induces cell activation and the release of proinflammatory mediators upon binding of FPR2 [37]. On the other hand, FPR2 mediates anti-inflammatory and pro-resolving effects when activated by LXA4 and AnxA1 [13, 38]. Considering the chemical and metabolic liability of LXA4, identification of new molecules able to activate FPR2 and mimic the pro-resolving effects of LXA4 is an important strategy to develop a therapy for AD.

Recently, we identified the new FPR2 agonist MR-39, which showed neuroprotective properties in an in vitro model of neuroinflammation. MR-39 had promising pharmacokinetic properties: good metabolic stability in rat microsomes (*t*_1/2_ = 48 min), good passive diffusion (*P*_app_ = 2512 nm/s), and good permeation rates (2.6) in hCMEC/D3 cells, an in vitro model of the blood–brain barrier [29]. In addition, MR-39 was able to reduce nitric oxide (NO) release and to attenuate the production of TNF-α and IL-1β in rat primary microglial cells stimulated with LPS [29].

As a follow-up in the present study, we investigated anti-inflammatory and pro-resolving potential of MR-39 ex vivo (i.e., in organotypic hippocampal cultures obtained from WT and KO FPR2 mice stimulated with Aβ_1-42_) and in vivo in double transgenic amyloid precursor protein/presenilin1 (APP/PS1) mice, a well-accepted model of AD.

Hippocampal organotypic cultures (OHCs) have significant advantages over the use of primary cell cultures or cell lines because they maintain intact hippocampal architecture and functional interactions between brain cells and the neuroimmune and endocrine systems, which allows to assess the effect of the tested compounds on both neuronal and glial cells [34, 39, 40] Considering that FPR2 is expressed on microglial cells [27, 41, 42] astrocytes and hippocampal neurons [43], OHC represents a valid ex vivo model to study the role of this receptor in the inflammatory processes related to AD.

Variable influence of Aβ treatment in OHCs suggested initial assessment of the impact of both fibrillar and oligomeric Aβ_1-42_ on cell viability in OHCs obtained from WT and KO FPR2 mice. In present study, we observed that the treatment with fibrillar but not oligomeric Aβ_1-42_ potentiated LDH release. In line with our observation the data reported by Richter et al., [42] demonstrated that application of Aβ_1‐42_ oligomer‐enriched preparations did not result in cell death in OHCs. In addition, other studies showed that treatments with various Aβ oligomers (Aβ_1‐28_, Aβ_25‐35_, and Aβ_1‐40_) failed to trigger neuronal cell death in OHCs [44–46]. Based on the available data, it may be speculate that in OHC’s model, which retain functional interaction between neuronal and glial cells, Aβ_1-42_ toxicity is strongly affected by microglia. Consistently, the use of clodronate to remove microglia showed that, Aβ_1-42_ toxicity is enhanced. Moreover, depleting microglia resulted in more significant Aβ_1-42_ deposition [41]. On the other hand, fibrillar Aβ_1-42_ is the key mediator of AD and is processed from the amyloid precursor protein (APP). Aβ_1-40_ and Aβ_1-42_ are the most common fibrillar Aβ isoforms; however, Aβ_1-42_ is the major constituent of senile plaques and is more prone to aggregation than Aβ_1-40_ [47]. Although we observed that fibrillar Aβ_1-42_ upregulate the LDH release in cultures obtained from both WT and FPR2 KO mice, MR-39 attenuated cell death evoked by fibrillar Aβ_1-42_ treatment only in hippocampal cultures from WT mice that suggest that MR-39 action is FPR2-dependent. The toxicity in OHCs is highly dependent on the applied dose and isoforms of Aβ [30, 40, 48], which limits its toxicity not only in a FPR2-dependent way but also in activating other receptors and pathways of intracellular signal transmission. Moreover, Aβ toxicity is mediated by secretion of neurotoxic reactive oxygen species (ROS), oxidative stress, and/or inflammatory mediators released from glial cells. Although our initial experiments demonstrated the toxic effect of fibrillar Aβ_1-42_ on the viability of the cells, we did not detect statistically significant effect of Aβ on NO release, which undoubtedly requires further studies.

Next, we studied the influence of MR-39 on the immune response evoked by fibrillar Aβ_1-42_ by assessing the effect of the agonist on the cytokine profile in OHCs from WT and KO FPR2 mice. Under basal conditions, TNF-α was upregulated in OHCs from KO FPR2 mice compared with that in WT cultures. Aβ_1-42_ treatment induced an increase in the secretion of TNF-α in both WT and KO FPR2 hippocampal cultures, and this increase was attenuated by MR-39 in WT cultures. Moreover, this effect was blocked by pre-treatment of OHCs from WT mice with the FPR2 antagonist WRW4, indicating that the anti-inflammatory effect of MR-39 is mediated by interaction with FPR2. Thus, we suggest that MR-39 lowers the TNF-α levels increased by Aβ_1-42_ treatment and can, at least in part, reduce neuronal death and the consequent LDH release, as detected in the present study. Similar results were observed in the case of the release of IL-1β. MR-39 was able to decrease the production of IL-1β induced by Aβ_1-42_ treatment, and this effect was abolished by pre-treatment of OHCs with the antagonist WRW4 in WT cultures. We did not detect an impact of MR-39 on the enhanced levels of IL-6, suggesting that the beneficial potential of MR-39 is mainly related to the inhibition of microglial reactivity, which is the main source of IL-1β and TNF-α after Aβ_1-42_ stimulation.

As we have shown, the anti-inflammatory effect of MR-39 in WT OHC’s is mainly associated with the inhibition of proinflammatory response expressed as a suppression of IL-1β and TNF-αproduction. Thus, the anti-inflammatory effect of MR-39 is mainly directed, like other FPR2 ligands (e.g., LXA4, AT-LXA4) to inhibition of pro-inflammatory response induced by Aβ_1-42_ [28, 49]. At the same time, MR-39 and LXA4 itself do not induce pro-inflammatory FPR2 activation. Several hypotheses have been done to explain such complex pharmacology, including FPR2 dimerization or the binding of structurally diverse ligands at different regions of the FPR2. Recently, studies suggest that the divergent properties of FPR2 may be related to ligand-specific receptor conformational changes associated with the activation of different downstream signaling, a behavior known as “biased agonism” [14]. The biased signaling has been reported for several “small molecule” FPR ligands such as compound 43 and compound 17b [50, 51]. Interestingly, in line with “biased agonism hypothesis,” our preliminary data obtained in the primary microglia cultures demonstrated that the anti-inflammatory activity of MR-39 is mediated via suppression of ERK1/2 phosphorylation, as the favorable effect of endogenous FPR2 ligand lipoxin A4. This observation could at least in part explain the lack of pro-inflammatory action of both ligands, while maintaining the anti-inflammatory potential (Tylek et al., submitted).

Several studies have demonstrated that fibrillar Aβ_1-42_ can activate microglia through the interaction with FPR2 [31, 52]. On the other hand, the neuroinflammatory response induced by Aβ_1-42_ in FPR2 KO OHCs clearly points to the fact that Aβ_1-42_ can interact with other targets. Indeed, Aβ_1-42_ can bind toll-like receptors (TLRs) and induces pro-inflammatory cytokine transcription via MAPK/ERK and NFκB mediated signaling. The class B scavenger receptor CD36 and complex of TLR-4 with -TLR6 can also be involved in the activation of microglia and proinflammatory factors release by Aβ_1-42_ [53]. In addition, receptor for advanced glycated end-products (RAGE) can also detect glycated and truncated forms of Aβ_1-42_ leading to direct activation of NFκB. Nevertheless, these data do not contradict our observation that MR-39 may attenuate Aβ_1-42_ evoked neuroinflammatory response only in the presence of FPR2, since the main downstream signalling pathways affected by fibrillar Aβ_1-42_ include the MyD66/TRAF6/NF-kB pathway, whose activation leads to the production of TNF-α, IL-1β, and other inflammation-related factors [54]. Therefore, we assayed the levels of the proteins of the MYD88/TRAF6/NF-kB pathway in OHCs from both WT and KO FPR2 mice to determine the molecular mechanism of the anti-inflammatory effect of MR-39. We found that MR-39 treatment normalized an increase in MyD88 levels evoked by Aβ_1-42_, and this effect was blocked by WRW4 only in WT cultures. On the other hand, Aβ_1-42_ had a more pronounced impact on an increase in the phospho-p65/p65 subunit ratio in WT cultures compared with that in KO FPR2 cultures. Interestingly, MR-39 treatment diminished the stimulatory effect of Aβ_1-42_ on the NF-kB level in WT cultures, and WRW4 pretreatment suppressed the effect of MR-39. Therefore, the anti-inflammatory activity of MR-39 on TNF-α and/or IL-1β release was apparently linked to the inhibition of the MyD88/TRAF6/NF-kB pathway, and the presence of FPR2 was crucial for this effect.

Available data indicate that IL-1β is biologically inactive and must be cleaved and transformed into its bioactive form by enzymatic activity of caspase-1. This catalytic event takes place with participation of the pyrin domain containing 3 complex (NLPR3). After activation NLPR3 oligomerizes and recruits the adaptor ASC (apoptosis-associates speck-like protein containing CARD), which promotes caspase-1 (casp-1) activation leading to forming a complex of inflammasome 3 (NLRP3). Once activated NLPR3 induces the cleavage and secretion of the biologically active IL-1 and/or IL-18 [55, 56], which requires two functional signals. The first signal called priming process is provided by receptor-ligands or cytokines that induce transcriptional regulation of the genes for the proinflammtory cytokines and the components of NLRP3. Meanwhile, the second signal induced by a variety of molecules leads to the activation of caspase-1 [57].

Intriguingly, we observed that the treatment of OHCs with fibrillar Aβ_1-42_ induced upregulation of the level of NLRP3, ASC, and caspase-1 proteins. Moreover, our study is the first to demonstrate that MR-39 suppressed the NLRP3 inflammasome pathway, and this effect was abolished by WRW4 only in WT cultures. Importantly, the levels of ASC and caspase-1 in OHCs from KO FPR2 mice were higher than those in OHCs obtained from WT mice, suggesting enhanced cleavage of pro-interleukin IL-1β into active IL-1β and consequential elevation of the level of IL-1β in OHCs from KO FPR2 mice. Simultaneously, higher caspase-1 level observed in OHCs from KO FPR2 mice may pointed to the enhanced pyroptosis induced by fibrillar Aβ_1–42_, because caspase-1 is an important cleaving protein for Gasdermin D [58]. Additionally, the dysfunction of autophagy which often occurs during the AD development [59], and plays a vital role in the regulation of caspase-1 activity, should also be considered. Undoubtedly, the observation we made may have a complex substrate and require more studies.

Recent data highlighted the role of NLRP3 activation in AD pathogenesis [60], including tau pathology and the Aβ cascade hypothesis. In line, NLRP3 may act as a link between Aβ_1-42_ plaques and neurofibrillary tangles [48]. Furthermore, Aβ aggregates activate the NLRP3 inflammasome, resulting in a reduction in Aβ clearance and an increase in Aβ [61, 62]. The activated NLRP3 inflammasome also promotes tau hyperphosphorylation and tangle formation. In this context, our findings that MR-39 is able to suppress the NLRP3 inflammasome pathway are of crucial importance and may be a promising approach for the development of new therapeutic strategies for AD.

In addition to the favorable effect of MR-39 on the proinflammatory cytokine profile, the data of the present study showed that the absence of FPR2 limited the impact of MR-39 on IL-4 and IL-10 release after Aβ_1-42_ stimulation, indicating possible disturbances in the resolution of inflammation (RoI). In fact, IL-4 is mainly produced by astroglial cells, and its level is diminished in the brain tissue of transgenic mice overexpressing human APP [63]. The beneficial effect of IL-4 is also expressed by downregulation of the inflammatory response [64] or “alternative activation” of the immune cells. Moreover, IL-4 suppresses the TLR4 signalling cascade in microglial cells, therefore reducing the proinflammatory response of FPR2 [65].

Thus, an increase in the levels of anti-inflammatory cytokines induced by MR-39 treatment should be regarded as a beneficial pro-resolving effect that attempts to restore homeostasis in cytokine levels after Aβ_1-42_ administration but is detected only in WT OHCs. To the best of our knowledge, our study is the first to indicate that the lack of FPR2 affects the balance of pro- and anti-inflammatory responses in hippocampal cultures, shifting the profile of released cytokines towards proinflammatory mediators. Consistently, FPR2 is a “promiscous” receptor that can mediate both pro- and anti-inflammatory ligand-induced response. Further research is needed to elucidate the exact mechanism responsible for this fascinating phenomenon [14].

The promising effects of MR-39 on cell viability and cytokine profiles in OHCs suggested an investigation of possible functional relevance of MR-39 in AD, which was assessed by chronic administration of the compound to double-transgenic APP/PS1 mice. In agreement with our previously published results obtained in APP/PS1 mice [27], the number of neuronal cells in the cortex (layer V) and hippocampus (dentate gyrus) in APP/PS1 mice was diminished, and chronic administration of MR-39 tended to normalize these deficits. Importantly, the impact of MR-39 treatment on glial cells, important for the immune response [66], was more pronounced in the brain areas of APP/PS1 double-transgenic mice. In particular, prolonged microglial proinflammatory activation and secretion of IL-1β, TNF-α, NO, and other harmful factors form the background for neuroinflammation and neurotoxicity [67, 68] Hence, our observation that chronic MR-39 treatment of APP/PS1 mice diminished microgliosis in the cortex and showed a tendency to decrease microgliosis in the hippocampus appears to be important. The beneficial impact of chronic MR-39 treatment was evidenced by a decrease in the number of Iba1-positive cells and by the morphological data. On the other hand, astrogliosis in APP/PS1 mice, which we observed previously [27], was not influenced by MR-39 treatment. Therefore, the results of the present study indicated that MR-39 preferentially targeted microglial cells and modulated their reactivity in APP/PS1 transgenic mice. Moreover, the results of our ex vivo studies suggest that favorable effect of MR-39 on microglial cells in APP/PS1 mice can be related to higher expression of FPR2 on immune cells compared to that on astrocytes. This suggestion is in the agreement with the data obtained in the OHC model, in which we reported inhibition of the release of microglial proinflammatory cytokines by MR-39 only in cultures obtained from WT mice and not from KO FPR2 mice.

Although we did not assess the chronic impact of MR-39 on the cytokine profiles in APP/PS1 mice or on the MyD88/NFkB- and NLRP3-related pathways, based on data of other studies [61], it can be suggest that the action of MR-39 on microglial cells may also involve the modulation of these intracellular pathways. Our data indicated that chronic MR-39 administration modulated microglia but not astrocytes, while the persistent activation of microglial cells is known to be stimulated by the deposition of Aβ_1-42_ [47, 61]. Thus, we next explored the interplay between Aβ_1-42_ and microglial cells. Considering that aggregation of Aβ_1-42_ is a hallmark of AD, we analyzed the hippocampal plaque load. Importantly, our data indicated that chronic MR-39 treatment diminished the area of Aβ_1-42_ plaques. A more sophisticated analysis revealed that the potency of the beneficial effect of MR-39 did not depend on the size of Aβ_1-42_ plaques.

## Conclusions

Considering the complex role that FPR2 may play in the inflammatory response in AD, the present study investigated the impact of the FPR2 agonist MR-39 on Aβ_1-42_-induced neuroinflammation in ex vivo and in vivo models related to AD. We found that MR-39 was able to abolish Aβ_1-42_-induced changes in both models. In OHC, anti-inflammatory and pro-resolving effects of MR-39 were mediated by a reduction in the Aβ_1-42_-evoked release of proinflammatory cytokines and concomitant increase in the production of anti-inflammatory cytokines. Interestingly, the beneficial effect of MR-39 was apparently related to the inhibition of the MyD88/TRAF6/NFkB signalling pathway and NLRP3 inflammasome activation. In APP/PS1 mice, MR-39 administration improved neuronal survival and reduced microglial cell density and plaque load. However, despite the promising anti-inflammatory and pro-resolving role of MR-39 in AD reported in the present study, additional investigations are needed to clarify the regulatory mechanisms of these pathways, which may influence the functionality of MR-39 in various models of AD pathology.

## Data Availability

All data supporting the conclusions of this manuscript are provided in the text, figures and tables.
